# Burden, clinical outcomes and predictors of time to in hospital mortality among adult patients admitted to stroke unit of Jimma university medical center: a prospective cohort study

**DOI:** 10.1186/s12883-019-1439-7

**Published:** 2019-08-30

**Authors:** Ginenus Fekadu, Legese Chelkeba, Ayantu Kebede

**Affiliations:** 1 Department of Pharmacy, Institute of Health Sciences, Wollega University, P.O Box 395, Nekemte, Ethiopia; 2 School of Pharmacy, Institute of Health, Jimma University, Jimma, Ethiopia; 3 Department of Epidemiology, Institute of Health, Jimma University, Jimma, Ethiopia

**Keywords:** Stroke, Burden, Outcome, Mortality, Ethiopia

## Abstract

**Background:**

The global burden of stroke epidemiology is changing rapidly. Over the 1990–2013 periods, there was a significant increase in the absolute number of deaths and incident events of stroke. The burden of stroke varies in Ethiopia between regions and over time. Hence, this study was aimed to assess the burden, clinical outcomes and predictors of time to in hospital mortality among stroke patients.

**Methods:**

A prospective cohort study was carried at stroke unit of Jimma University Medical Center (JUMC) from March 10–July 10, 2017. The outcome of interest was mortality and time to death. Data was analyzed using SPSS version 20. Multivariable Cox regression was used to identify the predictors of in hospital mortality and time to death from hospital arrival. Predictor variable with *P* < 0.05 was considered statistically significant.

**Results:**

A total of 116 eligible stroke patients were followed over 4 months. The mean age of patients was 55.1 + 14.0 years and males comprised of 73 (62.9%). Stroke accounted for 16.5% of total medical admissions. Among the 116 patients with stroke, 91 (78.4%) were discharged alive making in hospital mortality rate of 25 (21.6%). The median time of in hospital mortality and length of hospital stay after admission of the patients were 4.38 days and 9.21 days, respectively. The prominent suspected immediate cause for in hospital mortality was increased intracranial pressure in 17 (68.0%) followed by respiratory failure secondary to aspiration pneumonia in 11 (44.0%) patients. Brain edema (AHR: 6.27, 95% CI: 2.50–15.76), urine incontinence (AHR: 3.48, 95% CI: 1.48–8.17), National Institute of Health Stroke Scale (NIHSS) > 13 during hospital arrival (AHR: 22.58, 95% CI: 2.95–172.56) and diagnosis of stroke clinically alone (AHR: 4.96, 95% CI: 1.96–12.54) were the independent predictors of time to in hospital mortality.

**Conclusions:**

The mortality rate of stroke in this setup was comparable with other low- and middle-income countries (LMICs). There is an urgent need to establish well equipped and staffed stroke units in the country in addition to strengthening the already existing one’s. Furthermore, future work must be designed to identify the barriers to improve stroke outcomes and recovery.

**Electronic supplementary material:**

The online version of this article (10.1186/s12883-019-1439-7) contains supplementary material, which is available to authorized users.

## Background

Stroke is an important health problem worldwide and pose huge burden on the community health purse as well as on patients and their relatives [1, 2]. The global burden and clinical outcome of stroke epidemiology is changing rapidly [3]. Globally according to American Heart Association (AHA) report of 2016, stroke accounts for 11.8% of total deaths and ranked second leading cause of death next to heart disease in 2013 [4]. First-time incidence of stroke occurs almost 17 million times per year worldwide which was approximately one every 2 s [5].

Stroke remains one of the most devastating and disabling of al cerebrovascular disease with significant amount of residual deficit leading to economic loss [6–8]. The burden of stroke is high and is not only attributable to its high mortality but also its consequent high morbidity and physical disability [7, 9, 10]. Globally One in six people have a stroke in their lifetime and patients younger than 50 years accounts for 5–10% of all stroke [2, 11].

The global burden of disease (GBD) study also indicated that 80% of stroke deaths occur in low and middle income countries (LMICs) [1], showing that the developing world carries the highest burden of stroke mortality, morbidity and stroke related disability [8, 12–14]. Due to change in public exposure to risk factors and inability to afford high cost of care, the poor are increasingly affected by stroke [2]. Major problems shared by many countries of developing are a lack of infrastructure, poor systems of health care, lack of effective programs to address risk factors, shortage of adequately trained manpower and lack other resources to combat the epidemic [15–17]. Moreover it remains uncertain if increased life expectancy and urbanization in some parts of African nations will shift the continent to higher chronic diseases in future years [18]. However, the contribution of various risk factors to the burden of stroke is unknown, particularly in LMICs [19].

The burden and outcome of ischemic and hemorrhagic stroke varies in Ethiopia between regions and over time periods [20]. Most deaths in Ethiopia occurred early after admission due to stroke related acute medical and neurological complications. Because of late presentation and poor standard of care the in hospital mortality is higher and majority of the patients were discharged with severe physical disability [10, 21]. Poor treatment and inadequate rehabilitation services during hospital discharge has a series implication especially in hemorrhagic stroke patients in terms of saving life due to its severe neurologic complications characteristics [1].

Despite the high burden of strokes worldwide, there is insufficient information on the current epidemiology, prevention, management and outcome of stroke in African countries and other LMICs [10, 16, 17, 22]. This paucity of information has limited research output and consequently the way to overcome this burden in developing countries [23]. Hence, this study was aimed to assess burden, clinical outcomes and predictors of time to in hospital mortality among adult patients admitted to stroke unit (SU) of Jimma university medical center (JUMC).

## Methods

A prospective cohort study was carried out at SU of JUMC found in Jimma city, south-west Ethiopia from March 10–July 10, 2017. All adult stroke patients diagnosed clinically or confirmed by imaging and admitted to stroke unit of JUMC during the study period were included. Those not willing to give an informed consent, changed diagnosis of stroke, who died before evaluation, stroke readmission, stroke transformation, undetermined stroke subtype and patients with transient ischemic attack (TIA) and hematomas were excluded.

### Outcome and validating methods

In hospital mortality after hospital admission of the patient was considered as primary outcome of the study. Patients were followed from hospital arrival until died in hospital or discharged from hospital. Death ascertainment was based on physician duty note along with suspected immediate causes of death. Length of hospital stay/ admission was calculated as the time gap from the patient admission to stroke unit until discharged or died in the hospital. In addition, there were different validating methods that measures important predictors of outcome of interest. Stroke severity was obtained as per by the National Institute of Health Stroke Scale (NIHSS) [21], level of consciousness was obtained by Glasgow Coma Scale (GCS) [24] and physical disability was measured using modified Rankin scale (mRs) [21, 25]. Collection of clinical endpoints and other needed parameters were performed daily from the time of patient hospital arrival until patient died in the hospital or discharged. The decision to perform different ancillary tests, laboratories, imaging and clinical history taking was left to the attending physician [26].

### Data collection tool and procedure

Two qualified nurses and one medical resident were trained with the data collection instrument and collected the relevant data. Data was collected using interviewer administered questionnaire and semi structured data extraction format from the medical records of the patients. Data collection tools was developed based on the previous literatures and using the WHO step wise approach to stroke surveillance [27]. Important histories used for the study was taken from the patient and/or caregivers in the language they understood. English, Afan Oromo and Amharic version of the questionnaires were utilized to collect the data. All relevant information about each patient such as sociodemographic characteristics, length of hospital stay, causes of mortality, outcomes and associated factors were recorded carefully. Five percent of the sample was pre-tested to check acceptability and consistency of data collection tool 2 weeks before the actual data collection. Data were collected on hardcopy and entered into an electronic database.

### Statistical analysis

Data was entered into Epi data version 3.1 and analyzed using SPSS version 20. Descriptive statistics such as proportions, means, medians, standard deviations and interquartile ranges were calculated to describe data. In hospital mortality rate was compared using Kaplan–Meier and log rank test. Cox regression was used to identify predictors of time to in hospital mortality. *P* value < 0.05 was considered as cut off point to select candidate variables on binary Cox regression. Multivariable Cox regression with backward stepwise approach was used to identify the independent predictors of time to stroke mortality. Interaction between covariates and types of strokes were tested. A *p*-value < 0.05 was considered statistically significant.

### Operational definitions

**Glasgow coma scale:** Helps to measure level of consciousness [24, 28]**.**

- Good GCS (13–15): Mild brain injury (alert).
- Moderate GCS (9–12): Moderate brain injury (drowsy).
- Poor GCS (≤8): Severe brain injury (unconscious).

**Modified Rankin scale (mRS):** A scale that indicates the level of handicap in a person and used for the evaluation of physical disabilities of the patients during discharge [21, 25, 27, 29].

- Grade 0: No symptoms at all
- Grade 1: No significant disability despite symptoms; able to carry out all usual duties and activities
- Grade 2: Slight disability; unable to carry out all previous activities, but able to look after own affairs without assistance
- Grade 3: Moderate disability; requiring some help, but able to walk without assistance
- Grade 4: Moderately severe disability; unable to walk without assistance, unable to attend to needs without assistance
- Grade 5: Severe disability; bedridden, incontinent, and requiring constant nursing care and attention
- Grade 6: Dead

**Categorized the modified Rankin scale (mRS) outcome** [11, 21, 30]:

- mRS: 0–2 (Mild disability/ good outcome/ independence)
- mRS: 3 (Moderate disability)
- mRS: 4–5 (Severe disability)
- mRS: 6 (Death)

**NIHSS**: helps to assess the severity of stroke and the intervals were defined as [21, 28].

- NIHSS 0–6: Mild
- NIHSS 7–12: Moderate
- NIHSS 13–20: Severe
- NIHSS ≥21: Very severe

## Result

During the study period among 756 medical admissions, stroke related admission accounted for 125 (16.5%). From the total admissions, 110 of them experienced in hospital mortality and stroke accounted for 26 (23.6%) of the in hospital mortality. Nine patients were excluded from the study for various reasons (Additional file 1: Selection of study participants). Of the 116 study participants included in the final analysis, 61 patients (52.6%) had CT scan of the brain performed while, 55 (47.4%) of patients were evaluated clinically alone for stroke. According to the CT scan findings, 30 (49.2%) patients were found to have infarction while, 31 (50.8%) had hemorrhagic stroke [31]. Overall, using WHO criteria, 60 (51.7%) of the patients had ischemic stroke (IS) while, 56 (48.3%) had hemorrhagic stroke (HS) [26, 28].

### Demographic and patients baseline characteristics

The mean age of the patients was 55.1 + 14.0 years (ranged: 23 to 96 years). Stroke in youngsters (age < 45 years) comprised of 22.4% of all patients and males comprised of 73 (62.9%) [26, 28, 32] **(**Table 1**).**

**Table 1:** Demographic and baseline characteristics of stroke patients admitted to stroke unit of JUMC from March 10–July 10, 2017

Demographic and baseline characteristics		Frequency (*n* = 116)	Percentage (%)
Age (years)	< 45	26	22.4%
	45–65	65	56.0%
	> 65	25	21.6%
Sex	Male	73	62.9%
	Female	43	37.1%
Residence	Rural	84	72.4%
	Urban	32	27.6%
Marital status	Married	104	89.7%
	Widow	11	9.5%
	Divorced	1	0.9%
Education status	Unable to read and write	42	36.2%
	Able to read and write, informal education	49	42.2%
	Elementary school (1–8)	17	14.7%
	Secondary school (9–12)	3	2.6%
	College/university or above	5	4.3%
Occupational status (over the last 1 years)	Agriculture / farmer	44	37.9%
	Homemaker/ housewives	41	35.3%
	Merchant	11	9.5%
	Retired	6	5.2%
	Government employee	5	4.3%
	Other own business work	5	4.3%
	Skilled/unskilled manual labor/ daily worker	4	3.4%
Body mass index (BMI) (kg/m^2^)	< 18.5 (underweight)	24	20.7%
	18.6–24.9 (normal)	74	63.8%
	25.0–29.9 (overweight)	18	15.5%
Approximated monthly income (Dollar)	None (dependent)	20	17.2%
	< 20	46	39.7%
	20–40	25	21.6%
	> 40	25	21.6%

### Outcome and discharge condition of the patients

From the total 116 stroke patients admitted, 91 (78.4%) patients were discharged alive making in hospital mortality of 25 (21.6%). From those discharged, 67 (57.8%) were discharged with improvement and 16(13.8%) were left against medical advice (LAMA) on self and family request. Eighty one (89.0%) patients were discharged to home, but the remaining 10(11.0%) were transferred/referred to other hospital/ ward /health facility due to other comorbid diseases.

The mean National Institute of Health Stroke Scale (NIHSS) of the patients during discharge was 10.32 + 5.8, which was higher in HS patients compared to IS patients (11.10 + 6.4 versus 9.75 + 5.3) without statistically significant difference (*P* = 0.28). Majority of the patients 43 (47.3%) had moderate NIHSS and only one patient had severe brain injury (GCS ≤8) during discharge.

The mean modified Rankin score (mRS) at discharge was 3.97 + 1.5 with statistically significant difference (*P* = 0.013) between IS and HS [IS = 3.63 + 1.38 and HS = 4.34 + 1.55]. During discharge, majority of the patients 44(37.9%) had severe physical disability (mRS 4–5) and all patients “discharged to die” were classified as having mRS = 5 (severe disability) at the time of discharge. Generally, the median length of hospital stay for all patients was 9.21 days (ranged: 0.29–39.01 days) and specifically it was 9.88 days and 8.49 days for IS and HS patients, respectively. Seventeen patients (14.7%) discharged within 3 days and 22 patients (19.0%) stayed for greater than 2 weeks after hospital admission **(**Table 2**).**

**Table 2:** Outcome and discharge conditions of stroke among adult patients admitted to Stroke unit of JUMC from March 10–July 10, 2017

Outcome and discharge conditions		Total patients (*n* = 116)	Ischemic stroke (*n* = 60)	Hemorrhagic stroke (*n* = 56)	*P* value (OR)
vital status of the patient during discharge (*n* = 116)	Improved	67 (57.8%)	42 (70%)	25 (44.6%)	–
	Dead	25 (21.6%)	7 (11.7%)	18 (32.1%)	0.010
	LAMA on self and family request	16 (13.8%)	8 (13.3%)	8 (14.3%)	0.350
	Not improved/ the same condition/static	4 (3.4%)	2 (3.3%)	2 (3.6%)	0.615
	Referred to higher facility	3 (2.6%)	1 (1.7%)	2 (3.6%)	0.332
	Worsened / residual motor deficit	1(0.9%)	0 (0%)	1(1.8%)	–
NIHSS at discharge (*N* = 91)	Mean + SD	10.32 + 5.80	9.75 + 5.32	11.10 + 6.40	0.275
	NIHSS 0–6 (mild)	23 (25.3%)	15 (28.3%)	8 (21.1%)	–
	NIHSS 7–12 (moderate)	43 (47.3%)	24 (45.3%)	19 (50.0%)	0.460
	NIHSS 13–20 (severe)	19 (20.9%)	12 (22.6%)	7 (18.4%)	0.890
	NIHSS ≥21 (very severe)	6 (6.6%)	2 (3.8%)	4 (10.5%)	0.173
GSC at discharge (*N* = 91)	Median	15.0	15.0	15.0	0.571
	Poor GCS (≤8)	1 (1.1%)	0 (0%)	1 (2.6%)	–
	Moderate GCS (9–12)	8 (8.8%)	5 (9.4%)	3 (7.9%)	0.828
	Good GCS (13–15)	82 (90.1%)	48 (90.6%)	34 (89.5%)	1.000
mRS at discharge (*n* = 116)	Mean + SD	3.97 + 1.5	3.63 + 1.38	4.34 + 1.55	0.013
	mRS: 0–2 (mild disability)	20 (17.2%)	13 (21.7%)	7 (12.5%)	–
	mRS: 3 (moderate disability)	27 (23.3%)	19 (31.7%)	8 (14.3%)	0.696
	mRS: 4–5 (severe disability)	44 (37.9%)	21 (35.0%)	23 (41.1%)	0.203
	mRS: 6 (death)	25 (21.6%)	7 (11.7%)	18 (32.1%)	0.016
Length of hospital stay (days) (*n* = 116)	Mean + SD (days)	9.21 + 6.82	9.88 + 7.47	8.49 + 6.03	0.276
	< 3 days	17 (14.7%)	6 (10.0%)	11 (19.6%)	–
	3.01–7 days	32 (27.6%)	18 (30.0%)	14 (25.0%)	0.167
	7.01–14 days	45 (38.8%)	25 (41.7%)	20 (35.7%)	0.160
	> 14 days	22 (19.0%)	11 (18.3%)	11 (19.6%)	0.360

*GCS Glasgow coma scale, *LAMA* Left against medical advice, *mRS* Modified Rankin score, *NIHSS* National institute of health stroke scale, *OD* Odds ratio, *SD* Standard deviation

### In hospital mortality of stroke patients

Compared to ischemic stroke patients, those with hemorrhagic stroke experienced higher in-hospital mortality [32.1% versus 11.7%] (*p* = 0.01). The median time lapse between in hospital mortality and admission was 4.38 days (ranged: 0.29–13.75 days). But, specifically for that of IS and HS it was 4.30 days and 4.41 days respectively. From those 25 patients who experienced in hospital death, ten patients (8.6%) were died within 3 days, 10 (8.6%) between 3 to7 days and 5 (4.3%) died after 1 week of hospital admission.

### Immediate causes and predictors of time to in hospital stroke mortality

The prominent suspected immediate causes for in hospital mortality forwarded by clinicians was increased intracranial pressure 17 (68.0%) followed by respiratory failure secondary to aspiration pneumonia 11 (44.0%) **(**Table 3**).**

**Table 3:** Immediate causes of in hospital mortality of stroke among adult patients admitted to stroke unit of JUMC from March 10–July 10, 2017

Immediate causes of death for in hospital mortality	Total patient (*n* = 25)	Ischemic stroke (*n* = 7)	Hemorrhagic stroke (*n* = 18)
Increased intracranial pressure (ICP)	17 (68.0%)	2 (28.6%)	15 (83.3%)
Respiratory failure secondary aspiration pneumonia	11 (44.0%)	4 (57.1%)	7 (38.9%)
Stroke itself (primary or other stroke)	3 (12.0%)	1(14.3%)	2 (11.1%)
Ischemic heart disease	2 (8.0%)	1(14.3%)	1(5.6%)
Intracranial hemorrhage	1(4.0%)	0 (0%)	1(5.6%)
Refractory status epilepticus / other Seizure	1(4.0%)	1(14.3%)	0 (0%)
Hypertensive encephalopathy	1(4.0%)	0 (0%)	1(5.6%)
Renal and hepatic diseases	1(4.0%)	0 (0%)	1(5.6%)
Other heart diseases	1(0.9%)	1(14.3%)	0 (0%)

On multivariable Cox regression analysis, development of brain edema during hospitalization, urine incontinence during hospital presentation, having NIHSS> 13 upon hospital arrival and diagnosis of stroke clinically alone were the independent predictors of time to in hospital mortality among stroke patients. The rate of in hospital mortality in patients who had developed brain edema/ increased intracranial pressure (ICP) during hospitalization was 6.27 times more than those patients without brain edema (AHR: 6.27, 95% CI: 2.50–15.76). Similarly the risk (rate) of in hospital mortality in patients who had urine/bladder incontinence during initial hospital presentation was 3.48 times more than those without urine incontinence (AHR: 3.48, 95% CI: 1.48–8.17). Additionally, the risk (rate) of in hospital mortality in patients who had severe to very severe NIHSS (> 13) during hospital arrival was 22.58 times more than those patients with mild to moderate NIHSS (< 13) during hospital arrival (AHR: 22.58, 95% CI: 2.95–172.56. Finally, the risk (rate) of in hospital mortality in patients whose stroke was diagnosed clinically alone without imaging confirmation was 4.96 times more than those whose stroke was diagnosed by imaging modalities (AHR: 4.96, 95% CI: 1.96–12.54) (Table 4**).**

**Table 4:** Predictors of in hospital mortality among adult stroke patients admitted to stroke unit of JUMC from March 10–July 10, 2017

Variables		Dead	alive	CHR 95%CI	*P* value	AHR 95%CI	*P* value
GCS of the patient on hospital arrival	≤8	10	7	18.44 (5.06–67.24)	< 0.001		
	9–12	12	21	8.53 (2.41–30.22)	0.01		
	13–15	3	63	1.00			
NIHSS at hospital arrival	> 13	24	49	15.06 (2.04–111.37)	0.008	22.58(2.95–172.56)	0.003*
	< 13	1	42	1.00			
Urine/ bladder incontinence	Yes	16	28	2.56 (1.13–5.80)	0.024	3.48 (1.48–8.17)	0.004*
	No	9	63	1.00			
Comatose presentation	Yes	7	4	6.21 (2.56–15.07)	< 0.001		
	No	18	87	1.00			
Ways of stroke diagnosis	Clinically	17	38	3.37 (1.43–7.94)	0.005	4.96 (1.96–12.54)	0.001*
	Imaging	8	53	1.00			
Type of the stroke	Hemorrhagic	18	38	3.05 (1.27–7.31)	0.012		
	Ischemic	7	53	1.00			
Brain edema complication	Yes	18	17	6.93 (2.90–16.63)	< 0.001	6.27 (2.50–15.76)	< 0.001*
	No	7	74	1.00			
Swallowing difficulty	Yes	6	7	3.02 (1.19–7.64)	0.02		
	No	19	84	1.00			
Aspiration pneumonia complication	Yes	11	12	3.50 (1.60–7.72)	0.002		
	No	14	79	1.00			
Previous history of medication	No	16	39	2.29 (1.01–5.20)	0.047		
	Yes	9	52	1.00			

*AHR* Adjusted hazard ratio, *CHR* Crudes hazard ratio, *GCS* Glasgow coma scale, *NIHSS* National institute of health stroke

*Statistically significant at *p* < 0.05

**Fig. 1:**
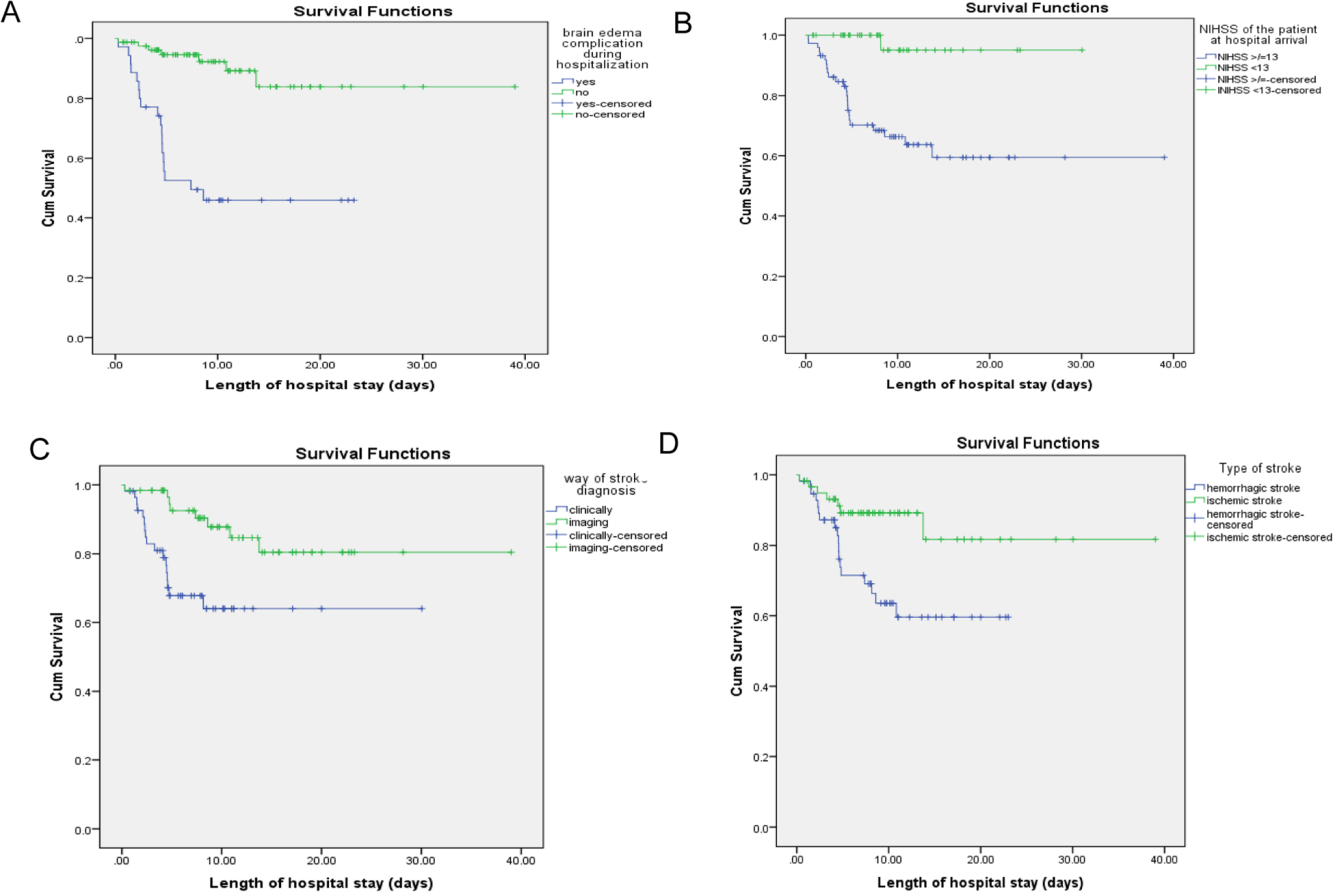
Survival probability curves derived from Log rank Kaplan Meier in hospital mortality and brain edema complication (**a**), NIHSS of patient during hospital arrival (**b**), way of stroke diagnosis (**c**) and type of stroke (**d**)

Survival probability curves derived from Log rank Kaplan Meier in hospital mortality with different factors was shown **(**Fig. 1**).**

## Discussion

In this study stroke accounted 16.5% of total medical admissions and 23.6% of the total medical cases of in hospital mortality. This admission rate was higher than findings from Gambia in which stroke patients constituted 5% [33] and in southwestern Nigeria made up of 4.5% medical admission [34]. However, this finding was in agreement with previous study conducted by Deresse B and Shaweno D in Hawasa in which stroke accounted for 13.7% of all medical admissions [21]. The elevated number of stroke admissions in Ethiopia might be due to lack of awareness, poor risk factor control and being hospital based study with referral bias.

From the total stroke patients admitted, 91(78.4%) patients were discharged alive and around one fifth (21.6%) of them experienced in hospital stroke mortality. From those discharged alive, more than half (57.8%) of the patients were discharged with improvement which was lower as compared to study by Masood et al in Pakistan (91%) [35], Jowi et al in kenya (93.8%) [36] and Tirschwell et al in Vietnam (65.8%) [16], but higher than study done by Gebremariam et al in Ethiopia (47.9%) [37]. On the other hand, our finding was comparable with study done in Ethiopia by Greffie et al in which 59.18% of the patients were discharged with improvement [10]. The outcome of the patients during discharge might vary with the severity of stroke, set up of the hospital, complications, comorbidities and experts available in caring of the patients.

The median length of hospital stay was 9.21 days which was shorter than previous studies by Walker et al 19 days [33], Jowi et al 12.5 days [36], Greffie et al 13 days [10], Gebremariam et al 11 days [37] and De Carvalho et al 15.4 days [38]. There are couple of reasons for the shorter length of hospital stay of stroke patients in our study setup. First, some patients were rapidly improved and discharged because they get better quality of care during the early phase at the unit compared to other wards in the hospital. Secondly, some patients died rapidly, some other left against medical advice (LAMA) and the remaining discharged with medical advice without improvement due to small bed number of the unit. With this regard, if the patient stayed longer than other patients and any improvement was observed, the bed was left for new stroke patients. Contrary to this, few patients were stayed in hospital for greater than 3 weeks. Multiple comorbidities, failed to show improvement and delayed in complimentary evaluations were some of the most feasible explanations for the prolonged length of hospital stay. This delayed evaluation will not only significantly increases the costs of stroke care, but also increases the risks of infection, other complications and recurrence in patients with suboptimal treatment and evaluation.

The in-hospital mortality of stroke patients (21.6%) was comparable with the study done by De Carvalho et al in Brazil 20.9% [38] and Desalu et al in Nigeria 23.8% [34]. However, it was higher as compared to study done by Deresse et al in Ethiopia 14.7% [21], Tirschwell et al in Vietnam 6.5% [16], Masood et al in Pakistan 9% [35], Gebremariam et al in Ethiopia 12.0% [37], Greffie et al in Ethiopia 13.3% [10] and Jowi et al in kenya (5%) [36]. But, the in hospital mortality rate in this was lower than the study done by Damasceno et al in Mozambique which was 33.3% [39], Atadzhanov et al in Zambia 40% [22] and Walker et al in Gambia 57% [33]. This difference could be due to different ways of stroke diagnosis, types of stroke, treatment approaches, comorbidities, complications and in hospital patient care.

The prominent immediate causes of death suspected by clinicians were increased intracranial pressure and respiratory failure secondary to aspiration pneumonia, which complies with other studies particularly conducted in Ethiopia [10, 21]. Furthermore, study done in Arabian Gulf countries reported that both neurologic and systemic complications accounted 63% of in hospital mortality [40]. However, it was different from study conducted by Walker et al in Gambia in which the most immediate cause of death was the initial stroke itself in 61% of patients [33]. The difference might be due to difference in physician’s assessment and prediction based on comorbidities as well as complications that were developed by the patients at the end of their life. Early identification and management of complications such as increased ICP and aspiration pneumonia will save life of some patients.

Generally, in hospital mortality rate of stroke in current study was higher than reports from western counties, but quite similar to SSA studies. This difference might reflect the limited access to hospital care, limited staffing, shortage of facilities for diagnosis, lack of necessary therapy and insufficient number of hospital beds for prolonged period of care in LMICs including Ethiopia. In addition to this, culturally some caregivers belief that patients should die at their home of origin where they spent most of their lives with family members around and caring for them. Absence of treatment with thrombolytic and the low frequency of treatment with antiplatelet agents for patients with ischemic stroke as well as lack of evaluation with neuroimaging with suboptimal care might be the other explanation for increased mortality of stroke patients in LMICs. Targeted interventions that reduce and control risk factors could substantially reduce the burden of stroke in SSA [19].

The median survival time for patients who died in hospital was 4.38 days which was shorter as compared to study by Walker et al 7.5 days [33], Greffie et al 6 days [10] and Damasceno et al 6 days [39]. However it was relatively comparable with the study done by Deresse and Shaweno in Ethiopia reporting that the median survival time of the patients was 4.5 days after hospital admissions [21]. The high mortality rate in this study during the first one-week (17.2%) might be due to acute complications developed among patients such as raised intracranial pressure and aspiration pneumonia.

Brain edema, urine incontinence, NIHSS> 13 during hospital arrival and diagnosis of stroke clinically alone were the independent predictors of time to in hospital mortality. Except stroke severity, other factors were not reported on study conducted by Atadzhanov et al in Zambia [22]. In this study increased NIHSS was associated with stroke severity constituting decreased level of consciousness. High NIHSS score as a predictor of mortality was consistent with previous study by Deresse and Shaweno in Ethiopia [21]. However, according to study by Sweileh et al stroke subtype was one independent predictor of in-hospital mortality among stroke patients [41].

In current study, increased ICP (brain edema) was one predictor of in hospital mortality unlike study by Mamushet et al in Ethiopia in which mortality was not significantly associated with increased intracranial pressure [42]. We believe that the number of in hospital complications were a reflection of the severity of stroke attack and it was an independent predictor of in-hospital mortality. The difference in predictors of in hospital mortality might be due to sample size, study design, significance value considered and eligibility criteria of the patient.

The in hospital mortality was higher for hemorrhagic stroke compared to ischemic stroke patients that complies with previous study findings [21, 22, 24]. Similar to study done by Das et al early onset mortality was common in hemorrhagic stroke, where late mortality was prevalent among ischemic stroke patients [43]. In contrary to this finding, study by Mamushet et al showed that mortality was significantly higher for ischemic stroke compared to hemorrhagic stroke cases (*P* = 0.049) [42]. The difference might be due to the study design, study population, stroke subtype and comorbidity of the cases. Similar to our finding, study by Deresse et al showed that the rate of stroke related mortality was not different by age and sex [21].

The major strengths of this study was its prospective study design and the enrollment of consecutive patients which allowed us for collection of reliable data on time-varying prevalence of multiple variables. We used core and supplementary ascertainment strategies, combined with an independent direct assessment, to achieve recommended gold-standard findings. Inclusion of first-ever and recurrent stroke cases during the study period would provide a more accurate reflection of the burden of stroke.

The study provided a preliminary database on mortality and functional outcomes among stroke patients during discharge which can pave the way for stroke management strategies. The degree of the neurologic deficit on discharge was evaluated based on functional status score, unlike in most of other studies which was categorized into those with and those without neurologic deficit. We have also performed a detailed NIHSS assessment allowing us to evaluate for determinants of outcome in series of patients with stroke. In addition, we have used survival analysis method with competing risk that allowed us to estimate the risks of stroke mortality.

The study was associated with some limitations. First, this study was a hospital-based study rather than large community based study. Hospital based study may not reflect true picture of the stroke as extremely critical patients died before hospitalization and mild cases may have not reported to hospital. Additionally, hospital based study is subjected to referral bias, as most of the acute stroke patients’ visit our hospital only from the south western part of Ethiopia directly without any selection. These referral bias, single setup as well as convenience sampling approach used might not reflect the true burden and outcome of the stroke in our community. Hence, extrapolations and generalization to the rest of the community should be done with caution. Even though the study was hospital based, having only one referral center might probably reflect the actual magnitude of stroke in our country. As well as, the mean age, the proportion of young adults, male predominance, incidence and mortality indices of our data were quite similar to other stroke epidemiological studies.

Secondly, etiologic investigation for stroke was infrequently performed due to the lack of systematic cardiological examinations and brain imaging. It was evident in this study that about half of the patients were diagnosed clinically alone for stroke. Diagnostic investigations were undertaken on the basis of the subjective findings indicating inadequate workup and hence, possible underestimation. Finally, the sample size was small hampering the analysis of some prognostic indicators due to the short recruitment period and lack of resources. Indeed, a prospective community-based cohort design will be required thousands of stroke-free subjects who need to be followed up for several years to know the outcome of patients.

## Conclusions

During discharge, majority of the patients were alive and discharged with improvement. The mean NIHSS of the patients during discharge was moderate, but majority of the patients had severe physical disability. Stroke mortality was high in our setup. The in-hospital stroke mortality was higher for hemorrhagic stroke and the prominent immediate cause for in hospital mortality was increased intracranial pressure and respiratory failure to secondary aspiration. Development of brain edema, urine incontinence NIHSS> 13 during hospital arrival and diagnosis of stroke clinically alone were the independent predictors of time to in hospital mortality.

The following points were forwarded as strategies to improve the stroke outcome. The non-governmental organizations and other non-profit organizations that work in areas of non-communicable diseases should focus towards the current debilitating conditions of stroke in SSA including Ethiopia through better funding of the health care system to improve the quality of care. Also there is an urgent need to establish well equipped and staffed stroke units in the country in addition to strengthening the already existing one’s.

Additionally, access to stroke experts, neurologists, rehabilitation therapists and other health care providers as well as availability of well-equipped diagnostic instruments are necessary to improve therapeutic strategies and reduces mortality. Finally, future work must be designed to identify the barriers to improve stroke outcomes and recovery. With this, prospective community based longitudinal studies are required to identify burden, risk factors and outcomes of stroke.

## Additional file

Additional file 1: Selection of study participants (DOCX 76 kb)

## Data Availability

The datasets used and/or analyzed during the current study are available from the corresponding author on reasonable request.

## Abbreviations

AHR: Adjusted hazard ratio
AOR: Adjusted odds ratio
GBD: Global Burden of Diseases
GCS: Glasgow coma scale
HS: Hemorrhagic stroke
IS: Ischemic stroke
JUMC: Jimma University Medical Center
LMICs: Low and middle income countries
NIHSS: National Institute of Health Stroke Scale
SSA: Sub Saharan Africa
SU: Stroke unit
WHO: World Health Organization
