# Supplementary material for: Burden, clinical outcomes and predictors of time to in hospital mortality among adult patients admitted to stroke unit of Jimma university medical center: a prospective cohort study
Source: BMC Neurol. 2019 Aug 30;19:213. doi: 10.1186/s12883-019-1439-7 (PMC6716869; doi:10.1186/s12883-019-1439-7)
Supplement: Supplementary file 1 — Selection of study participants (DOCX 76 kb) [file 12883_2019_1439_MOESM1_ESM.docx]

**Additional File 1: Selection of study participants**

Total patients visited the emergency and maternity ward during the study period suspected and confirmed to be stroke: 146 patients

**Excluded study participants in EOPD: 21**

- TIA or improved in the emergency: 6
- Hematoma linked to surgical ward: 3
- Other medical condition: 2
- Lost to follow up: 1
- Left against medical advice: 3
- Referred to other hospital: 2
- Died before evaluation: 2
- Normal imaging: 2

Total patients admitted to stroke unit within 4 months: 125

Excluded from the study: 9 patients

Fulfilling inclusion criteria

- TIA: 2
- Transformation:1
- Non responsive: 0
- Undetermined stroke clinically: 1
- Hematoma: 2
- Normal imaging:2
- Other medical case (epilepsy): 1
- Stroke readmission: 0

Clinical alone based diagnosis

Imaging based diagnosis (CT scan)

ICH: 23

ICH: 28

SAH: 2

IS: 30

SAH: 3

IS: 30

HS: 56

IS: 60

Exposed group: 56

Non-exposed group: 60

Total patients included in the final analysis: 116

* CT: computed tomography, EOPD: Emergency outpatient department, IS: ischemic stroke, HS: hemorrhagic stroke, SAH: subarachnoid hemorrhage, ICH: Intracerebral hemorrhage, TIA: transient ischemic attack

**Fig:** Selection of study population of eligible adult stroke patients admitted to stroke unit of JUMC.
